# The Human Cell Atlas bone marrow single-cell interactive web portal

**DOI:** 10.1016/j.exphem.2018.09.004

**Published:** 2018-09-21

**Authors:** Stuart B. Hay, Kyle Ferchen, Kashish Chetal, H. Leighton Grimes, Nathan Salomonis

**Affiliations:** a Division of Biomedical Informatics, Cincinnati Children’s Hospital Medical Center, Cincinnati, OH, USA; b Department of Pediatrics, University of Cincinnati School of Medicine, Cincinnati, Ohio, USA; c Division of Immunobiology and Center for Systems Immunology, Cincinnati Children’s Hospital Medical Center, Cincinnati, Ohio, USA; d Division of Experimental Hematology and Cancer Biology, Cincinnati Children’s Hospital Medical Center, Cincinnati, OH, USA; e Department of Biomedical Informatics, University of Cincinnati, Cincinnati, OH, USA

## Abstract

The Human Cell Atlas (HCA) is expected to facilitate the creation of reference cell profiles, marker genes, and gene regulatory networks that will provide a deeper understanding of healthy and disease cell types from clinical biospecimens. The hematopoietic system includes dozens of distinct, transcriptionally coherent cell types, including intermediate transitional populations that have not been previously described at a molecular level. Using the first data release from the HCA bone marrow tissue project, we resolved common, rare, and potentially transitional cell populations from over 100,000 hematopoietic cells spanning 35 transcriptionally coherent groups across eight healthy donors using emerging new computational approaches. These data highlight novel mixed-lineage progenitor populations and putative trajectories governing granulocytic, monocytic, lymphoid, erythroid, megakaryocytic, and eosinophil specification. Our analyses suggest significant variation in cell-type composition and gene expression among donors, including biological processes affected by donor age. To enable broad exploration of these findings, we provide an interactive website to probe intra-cell and extra-cell population differences within and between donors and reference markers for cellular classification and cellular trajectories through associated progenitor states.

The advent of new innovative technologies for single-cell genomics provides nearly limitless opportunities for exploring tissue cellular variation at single-molecule resolution. Single-cell RNA profiling has already revealed hidden heterogeneity within presumed homogenous populations, novel intermediates, and developmental trajectories [1–5]. Although thousands of cells can be readily captured and profiled with these technologies, the cellular composition of in vivo cellular niches are complex, currently requiring selective strategies for isolation such as flow cytometry sorting and a priori defined surface markers to capture and profile sufficient depths of rare cell populations [3,5–8]. Unbiased approaches that evaluate cells within their unperturbed cellular niches provide a means to characterize the broad spectrum and in vivo frequencies of cell populations.

The Human Cell Atlas (HCA) and the National Institutes of Health’s Human BioMolecular Atlas Program (HuB- MAP) are ambitious efforts to build detailed molecular reference maps of all cell populations in the human body [9]. The creation of an HCA is essential for accurate comparison of tissues exhibiting disease with comparable healthy tissues in order to enable translational discoveries. Although these projects will ultimately integrate complementary high-resolution imaging data for RNA and protein for cells in their intact niches, current efforts have been focused on single-cell genomics analyses of these tissues for tens of thousands of cells from diverse, presumably healthy, donors. These cells include potential multipotent progenitor cells that can manifest as mixed-lineage patterns of gene expression at a single-cell level [3,10]. Such mixed-lineage states reflect the molecular priming of different developmental potentials by coexpressed alternative lineage determinants that are challenging to identify from datasets of only one or a few individuals. The existence of such cell states remains controversial, as do the trajectories in which diverse hematopoietic progenitors specify. Therefore, very large and unbiased surveys of unperturbed bone marrow in a diverse cross-section of the human population are necessary to resolve these challenges. Given the complexity of the data produced through an HCA, easy-to-use and comprehensive analytical interfaces are necessary to promote the reuse of these data and the initial evaluation of hypotheses by the broader, noncomputational research community. Beyond significant efforts by the HCA consortium to create such resources, broad development of tools to provide new and deep insights into these datasets are necessary to exploit such data to reach its full potential.

Here, we present a comprehensive web portal to navigate data from initial HCA data production for over 100,000 human bone marrow cells spanning eight individual donors (http://www.altanalyze.org/ICGS/HCA/Viewer.php). Our deep analysis of molecular and cell heterogeneity was successful in resolving 35 transcriptionally coherent cell populations associated with diverse, previously defined committed cell lineages, potentially novel progenitors, and cellular trajectories. These include frequently occurring novel progenitor populations undergoing mixed-lineage priming analogous to recently characterized progenitors in the mouse [3]. Resulting novel populations and associated markers for distinct cell populations represent new reference sets and hypotheses to be explored in downstream studies by the larger research community through our provided resource. This portal allows users to evaluate empirically observed or custom population markers across diverse progenitor and specified cells and cellular maturation trajectories. We believe the application of such a resource will be broad by enabling the evaluation of individual genes in both common and extremely rare cellular compartments across and between diverse donor specimens. As an example, we demonstrate the power of this database through the comparison of the initial large-scale release of HCA bone marrow samples according to donor sex and age to resolve novel heterogeneity affecting stem cell gene expression.

## Methods

### Data acquisition and preprocessing

Bone marrow small conditional RNA-sequencing (scRNA- Seq) data from eight reported healthy donors was obtained from the HCA Data Portal (https://preview.data.humancellatlas.org) as gene-level (Ensembl) unique molecular index (UMI) counts (Cell Ranger software, 10 × Genomics GRCh38 standard reference). For each donor, HCA investigators captured cells on eight separate 10 × Genomic Chromium ports. Although, collectively, these data include data for over 270,000 cellular barcodes, we restricted analysis to barcodes with at least 200 genes expressed. Over 103,000 cells matching this filter were obtained, with all donors possessing at least 9,800 cellular barcodes from multiple HCA-provided captures per donor. As in other studies, gene expression was quantified as UMIs per gene divided by the total UMIs per barcode multiplied by a 10,000 (counts per ten thousand or CPTT), [11,12]. The majority of cells in this filtered dataset had less than 500 genes/barcode expressed (*n* = 73,246), with a third of the total (*n* = 38,771) possessing less than 300 genes/barcode. Batch effects were evaluated following single-cell alignment between donors (see below). Software and command-line options for these and downstream analyses are described in further detail in a separate document (Supplemental Document E1, online only, available at www.exphem.org).

### Cell population prediction and alignment

To identify cell populations independent of possible donor effects, we initially performed independent unsupervised cell population analyses for each donor using the Iterative Clustering and Guide-Gene Selection (ICGS) software workflow [3,12] available in AltAnalyze version 2.1.1. ICGS was run using the default options for 10 × Genomics datasets (euclidean HOPACH clustering, correlation cutoff > 0.3, protein-coding restricted) with the additional option to exclude cell cycle effects (conservative option) [3]. Cell cycle effects were later evaluated in downstream ICGS analyses of selected progenitor cell clusters. ICGS was restricted to genes with a CPTT > 1 in at least one cell. Following ICGS analysis of these scRNA-Seq data, expression medoids were calculated per obtained cell clusters for all reported cell-population-specific markers (MarkerFinder ICGS output). Medoids were used instead of centroids to minimize the potential impact of multiplets contained within each cluster. Marker genes can include those with a CPTT < 1 in each cell population. Donor cell cluster medoids were subsequently combined for all donors and clustered using the HOPACH library in R to obtain averaged medoid clusters and aggregated (average) based on pairwise similarity (Pearson rho > 0.9) to obtain 27 unique clusters (cellHarmonyMerge function in AltAnalyze). To combine the results from these donor-specific analyses without biasing to donor differences, we aligned all cells to these reference profiles using our recently developed algorithm k-nearest classification approach, which we call cellHarmony (https://github.com/nsalomonis/altanalyze/wiki/cellHarmony) [12]. cellHarmony was performed using a correlation cutoff of 0.7 to eliminate poor-quality barcode profiles and those with similarity to multiple references (e.g., doublets). MarkerFinder is designed to identify lineage-specific markers, making these markers more suitable for comparison between different donor samples (see batch effects evaluation below). Following cellHarmony, MarkerFinder was independently run on the derived set of 102,455 cells to derive revised markers.

### Cell population annotation

Cell type annotations were assigned using a custom gene-set enrichment strategy. Initial cell type predictions were produced from the software GO-Elite in AltAnalyze using its previously described cell and tissue marker gene database [3,13]. This database contains markers for dozens of selected sorted human cell populations in addition to in vitro-derived and bulk tissue samples [14]. This analysis suggests that nearly all populations are hematopoietic in origin or contaminating stromal cells. To assign more confident cell population annotations, we assembled a large hematopoietic and immune-specific compendium of 73 cell types. The sources of these cell-type profiles were: (1) human sorted bone marrow progenitor RNA-Seq (BluePrint) [15], (2) human sorted immune cell microarrays (GSE22886, GSM321573, GSE15907, GSE26928, and GSE23321), and (3) previously published peripheral blood mononuclear cell (PBMC) single-cell RNA-Seq data [16]. MarkerFinder was performed within each independent dataset to derive putative cell-population- specific markers for gene-set enrichment using GO-Elite (see gene associations in Supplementary Table E1, online only, available at www.exphem.org). This GO-Elite analysis was performed on the cellHarmony-classified cell populations following identification of new marker genes (MarkerFinder) (see enrichment results in Supplementary Table E1, online only, available at www.exphem.org). Secondary confirmation of these predictions was obtained by independent gene-set enrichment analysis using the web-based tool ToppFun [17] and literature marker evaluation.

### Focused progenitor analyses

To further resolve cellular heterogeneity among immune progenitors, cells from all donors for the 10 cell populations with the highest CD34 gene expression were selected and reanalyzed by ICGS as described above. These revised progenitor cell clusters were reanalyzed separately using MarkerFinder and our above cell population annotation workflow to assign labels. The MarkerFinder output from AltAnalyze was used as input for unsupervised trajectory analysis using the software SPRING [18]. The CD34^+^ separately evaluated cell populations were integrated with nonprogenitor populations for downstream comparison analyses (35 final cell populations). Mixed-lineage gene expression priming was assessed using a custom Python program (multiLineagePredict.py in AltAnalyze) using MarkerFinder cell population associated markers that were coincidentally present in at least one other cell population. Cells that coincidently expressed multiple alternative lineage markers within a given cell state were considered to exhibit mixed-lineage priming.

### Evaluation of batch effects

The contribution of technical effects from different 10 × Genomics captures of the same samples was evaluated in addition to donor-specific effects using data visualization and gene-set evaluation. Selective evaluation of the two cell populations with the greatest numbers of cells (naive T cells and neutrophils) was performed using principal component analysis (PCA) of all genes for all donors and batches using AltAnalyze. The contribution of donor-specific effects on all cell populations was evaluated using the independent unsupervised analysis software Seurat, using parameters recommended by the authors, but run without Canonical Correlation Analysis to assess donor differences for all genes. A restricted set of presumably donor un-confounded marker genes was selected from our 35 cell populations using the top 50 MarkerFinder genes per cell population (*n* = 1,869) (Supplementary Tables E2 and E4, online only, available at www.exphem.org). Seurat analysis was reperformed using these marker genes to assess their impact on population predictions. These results were empirically compared with donor of origin and cell-type specific assignments as outlined above.

### Donor comparisons

Differential expression analyses were performed on the averaged (pseudobulk) gene expression for the final 35 annotated cell populations for each donor using AltAnalyze. Differentially expressed genes were defined as those with an empirical Bayes moderated *t* test *p* < 0.05 (false discovery rate corrected) and > 1.5-fold between male and female donors for the same annotated cell population. Pearson correlation was used to identify genes with log2 expression correlated to donor age (log2 normalized) with a correlation coefficient more than 0.6 or less than −0.6. Gene-set enrichment analyses were performed with GO-Elite and ToppFun.

### Web portal development

The HCA Bone Marrow Viewer is built using a mixture of JavaScript, HTML, and PHP 7 to display scalable vector graphics built using the D3.js Javascript and library Plotly.js. The donor expression data for all 100,000 cells and associated genes are stored via a gene cell matrix in a locally accessible SQLite 3 database indexed by gene. Access for client-side users is facilitated through the standard PHP database extension for SQLite 3. Statistics and annotations are all calculated and retrieved server side using output buffering where necessary. For exploration of the tabular results, users can input up to five genes for a given request, which retrieves and parses the output in three formats: donor- specific, gene-specific, and cluster-specific. Each format is available for viewing from a boxplot (donor mean gene expression or all cell population normalized expression values) or as a frequency bar chart. To prevent compromise of client-side optimization, data are rendered once selected via the navigation sidebar. SPRING and UMAP results are viewed as 2D scatter plots derived through analysis from the respective programs.

## Results

**Figure 1.**
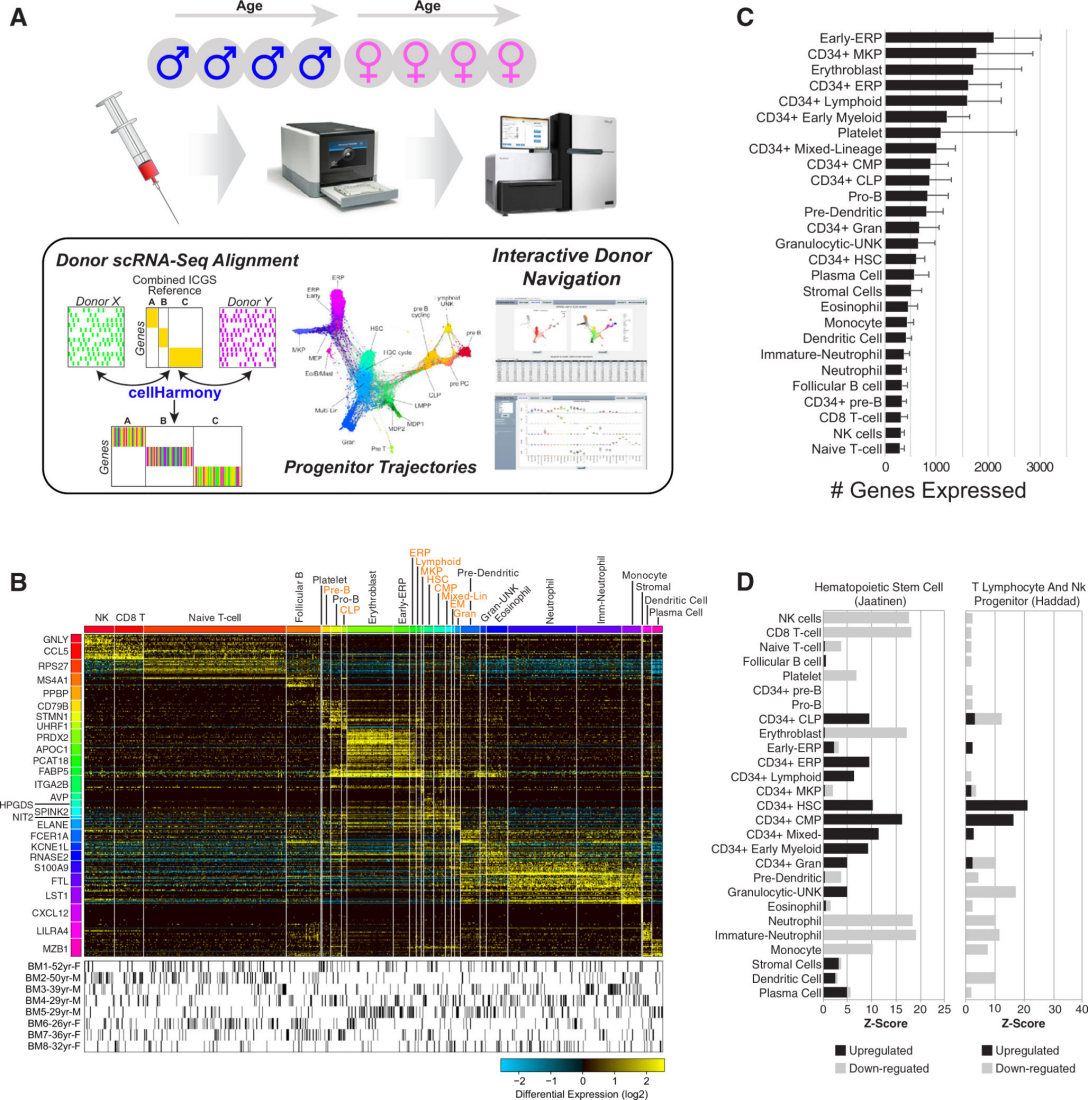
Integrated analysis of bone marrow hematopoietic cells from healthy donors. **(A)** Applied computational workflow to bone marrow cells processed by the HCA project. The three principle approaches applied are ICGS (unsupervised population analysis), cellHarmony (population alignment between donors), and SPRING (lineage trajectory analysis). An interactive web interface provides in-depth exploration of these data. **(B)** Integrated results heatmap for ~100,000 cells from all eight donors classified along 27 ICGS populations. Columns are cells and rows are genes. The top MarkerFinder gene for each population gene cluster is shown (left). Cells for each donor are shown below the heatmap. **(C)** Populations differing according to the average number of genes expressed. Error bars indicate standard deviation. **(D)** Statistical enrichment of gene sets from the Molecular Signature Database (Broad Institute) in the software GO-Elite corresponding to two frequently enriched progenitor categories as either upregulated or downregulated genes.

We set out to produce an integrated view of human hematopoietic diversity that encompasses differences among distinct bone marrow donors. To accomplish this goal, we developed an integrative computational strategy and data visualization workflow that allows for harmonization of different donor samples while exploring variation among them (Figure 1A). This workflow employs both previously described robust unsupervised approaches to delineate discrete and transitional cell populations (ICGS), align cells between donors (cell- Harmony), and assess differentiation trajectories (SPRING) (see Methods).

Given that distinct immune cell types are likely more or less transcriptionally quiescent than others, we initially selected cells with a relatively low expression for our initial analyses (200 genes/cell expressed = 103,141 barcodes) from over 270,000 posted barcode cell profiles provided in the first public release of the HCA. Importantly, the large majority of cells across all eight donors expressed less than 500 genes per cell (~2/3). To identify putative cell populations from this compendium, we applied our previously described algorithm ICGS, which can detect both discrete and mixed-lineage progenitor cell populations, separately for each donor [3,12]. Separate analysis of each donor was conducted to detect cell populations found in a single donor that might be missed in the combined analysis and to avoid donor effects. Aggregation and clustering of the cell population medoids identified 27 distinct cell populations across the eight donors, with unique cell-population- associated gene expression. To derive consistent cell population assignment using these reference cell signatures, we applied our recently developed k-nearest neighbor classification approach to all filtered donor cells [12]. As illustrated from the subsequent marker gene analysis of these aligned, all 27 cell populations were found to possess unique gene expression, with cells from all donors represented in each cell population (Figure 1B, Supplementary Table E2, online only, available at www.exphem.org). Initial cell population annotations (ICGS) predicted only one contaminating nonimmune cell population of stromal cells (0.2% of all cells).

Using an assembled library of over 70 annotated progenitor and immune cell types (bone marrow, PBMCs), we identified cell-population-associated gene clusters that were enriched in prior examined hematopoeitic subsets (see Methods and Supplementary Table E1, online only, available at www.exphem.org). The most frequent cell state identified is naive T cell (~25% of all cells), whereas the rarest is platelet (>0.1%), with only 65 cells identified. Megakaryocyte (MK) and erythroid lineage cells express the greatest number of genes (1600–2100), whereas natural killer (NK) cells, T cells, B cells, and neutrophils are the most transcriptionally quiescent among our 27 detected cell populations, with only 300–370 genes expressed on average (Figure 1C). In addition to well-defined mature cell populations typically found in peripheral blood, such as T cells, B cells, NK cells, eosinophils, monocytes, neutrophils, MK, and erythrocytes (ER), our analysis suggests the existence of diverse CD34^+^ progenitor subsets, including unknown cell populations similar to prior described progenitors (e.g., common myeloid progenitors, hematopoietic stem cells [HSC], ER progenitors [ERP], MK progenitors [MKP], and mixed-lineage progenitors). Consistent with these predictions, gene-set enrichment finds enrichment of stem and progenitor genes along with those associated with priming toward distinct lineages (Figure 1D, Supplementary Table E3, online only, available at www.exphem.org).

Through the independent analysis, integration, and classification of cell populations across donors, we expect our integrative analysis strategy to significantly reduce or eliminate possible donor-specific population effects. To first evaluate the presence of donor-specific effects, we applied PCA on cells associated with the two most frequent cell populations, naive T cells and neutrophils (Supplementary Figure E2, online only, available at www.exphem.org). As expected, donor- specific effects were clear; however, batch effects between the technical sample analyses were not evident. Using an independent unsupervised analysis tool (Seurat), driving specific donor effects were evident for multiple cell populations: neutrophils, immature neutrophils, and dendritic cells. However, restricted analysis of MarkerFinder-defined genes in Seurat vastly improved the association of the predicted populations via dimensionality visualization, providing additional evidence that our ICGS/cellHarmony strategy will highlight cell-population-specific rather than donor-specific gene expression differences.

**Figure 2.**
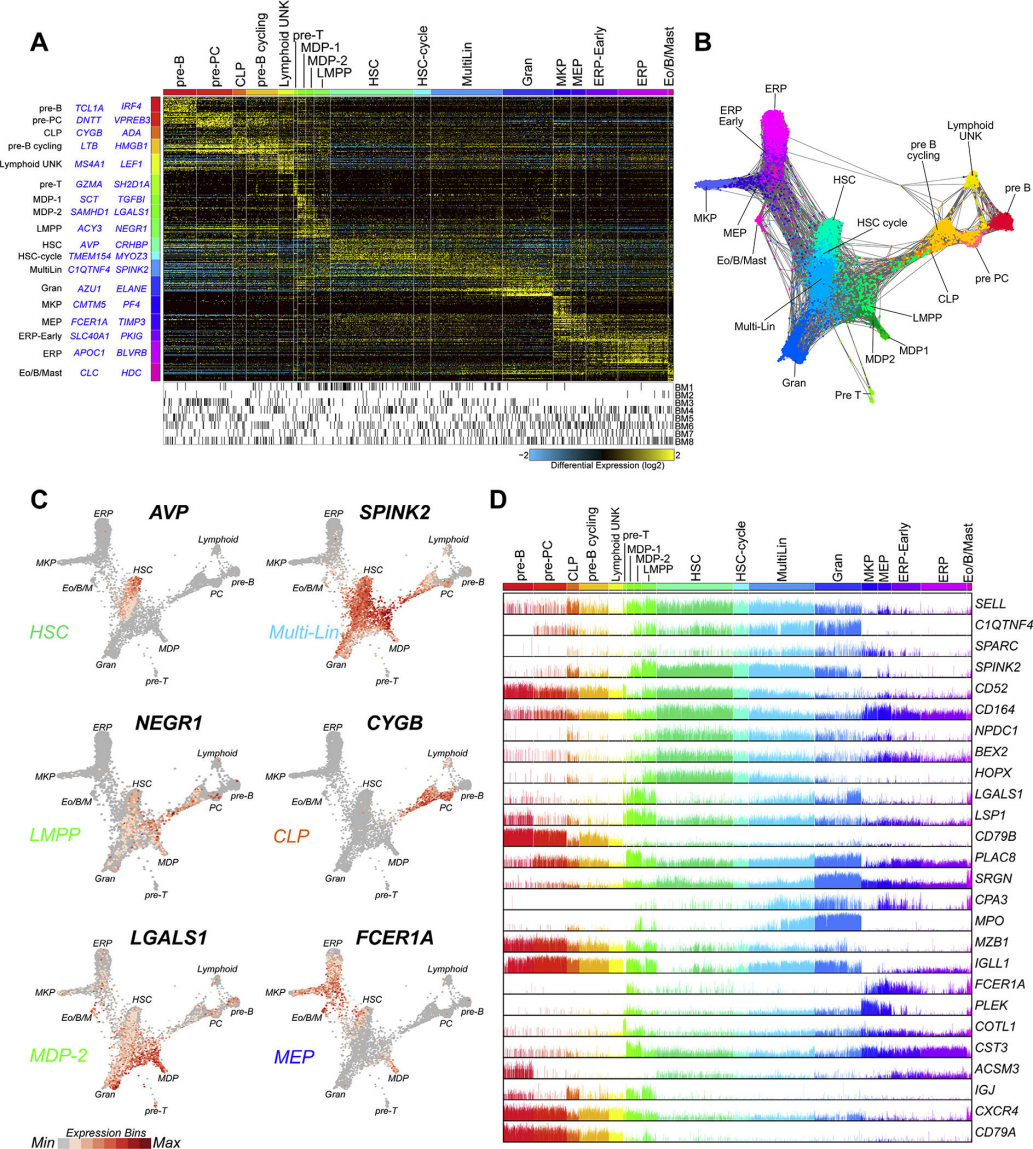
Identification of rare transitional progenitor populations. **(A)** ICGS analysis of ~12,000 combined donor CD34^+^ populations selected from Figure 1B. The top two MarkerFinder genes for each population gene cluster are shown (left). **(B)** SPRING analysis of all associated cells using ICGS population-associated genes. ICGS populations are notated by distinct colors and are labeled according to frequency. **(C)** Visualization of predicted transitional population (CLP, LMPP, Multi-Lin*, MEP, MDP)-associated marker genes in the SPRING graph according to normalized gene values (red=high expression bin, grey=no expression). **(D)** Bar plot of genes with evidence of multilineage priming in multiple ICGS populations, corresponding to transitional cell populations.

To better understand the diversity of early progenitors and presumptive stem cells within the bone marrow, we selected all cells in CD34^+^ cell populations and performed an independent unsupervised analysis (Figure 2A). Although CD34^+^ cell surface expression levels will not be accurately assessed by gene expression, in the absence of such data, we considered expression of CD34 in multiple donors in any cell population as a reasonable surrogate. As previously shown, the use of ICGS and particularly HOPACH clustering tends to emphasize transitions within and between cell populations, thus enabling more granular insights into cell heterogeneity [3]. From our starting set of 10 cell clusters, we resolved 18 CD34^+^ cell populations, which include those with graded gene expression differences indicating putative transition states and exceedingly rare progenitor states (eosinophil progenitors, *n* =119; T-cell progenitors, *n* = 82). We do not see evidence of cell clusters that result specifically from donor effects (Figure 2A, lower panel). In addition to gene-set enrichment, examination of the top marker genes assigned to cell populations agree well with prior literature (Supplementary Table E4, online only, available at www.exphem.org). These include the recently identified presumptive HSC markers AVP and EMCN [19,20]. To assess the hierarchical relationships from these cell transcriptomes, indicative of lineage trajectories, we analyzed the derived cell population markers for all CD34^+^ cells using a recently described force- directed layout method, SPRING [18] (Figure 2B). When gene expression for the presumptive transitional state markers are overlaid upon the SPRING network, we find that these markers exist at the junctions of distinct bipotential or multipotential specification branchpoints (e.g., ERP-MKP, MDP-early granulocyte progenitors [Gran]), in agreement with the ICGS predictions (Figure 2C). Further, whereas presumptive HSC, predicted from our hematopoietic gene marker library, display the least priming to other lineages, multiple populations display mixed-lineage priming, including presumptive megakaryocyte/erythroid progenitors (MEP), lymphoid-primed multipotent progenitors (LMPP), monocyte/dendritic cell progenitors (MDP), common lymphoid progenitors (CLP), eosinophil progenitors, and other multilineage progenitors (Multi-Lin) similar to those previously identified in mouse [3]. In each of these cell populations, mixed- lineage gene expression is evidenced by the joint expression of MarkerFinder cell population “restricted” genes expressed in at least 25% of cells in a different lineage for at least two other lineage programs (Figure 2D). These data suggest that presumptive HSC give rise to cycling HSC, which in turn produce Multi- Lin. Multi-Lin project into LMPP, Gran, MEP, and eosinophil progenitors, in agreement with our previously proposed model in mouse [3]. MEP are placed upstream of early-ERP and MKP and LMPP are upstream of CLP, MDP1, and MDP2. Whereas most putative trajectories are in agreement with well-established progenitor hierarchies, some SPRING trajectories such as pre-T cells emerging separately from B cells and plasma celllikely result from undersampling and/or a lack of “anchoring” in the graph from committed progeny. Further, although eosinophil progenitors appear to derive from two separate trajectories, one deriving from Multi-Lin and one deriving from MEP we presume that this ambiguity results from mutual GATA1 expression in MEP and eosinophils, making it appear that there are two origins to this granulocytic population. However, this requires experimental confirmation.

**Figure 3.**
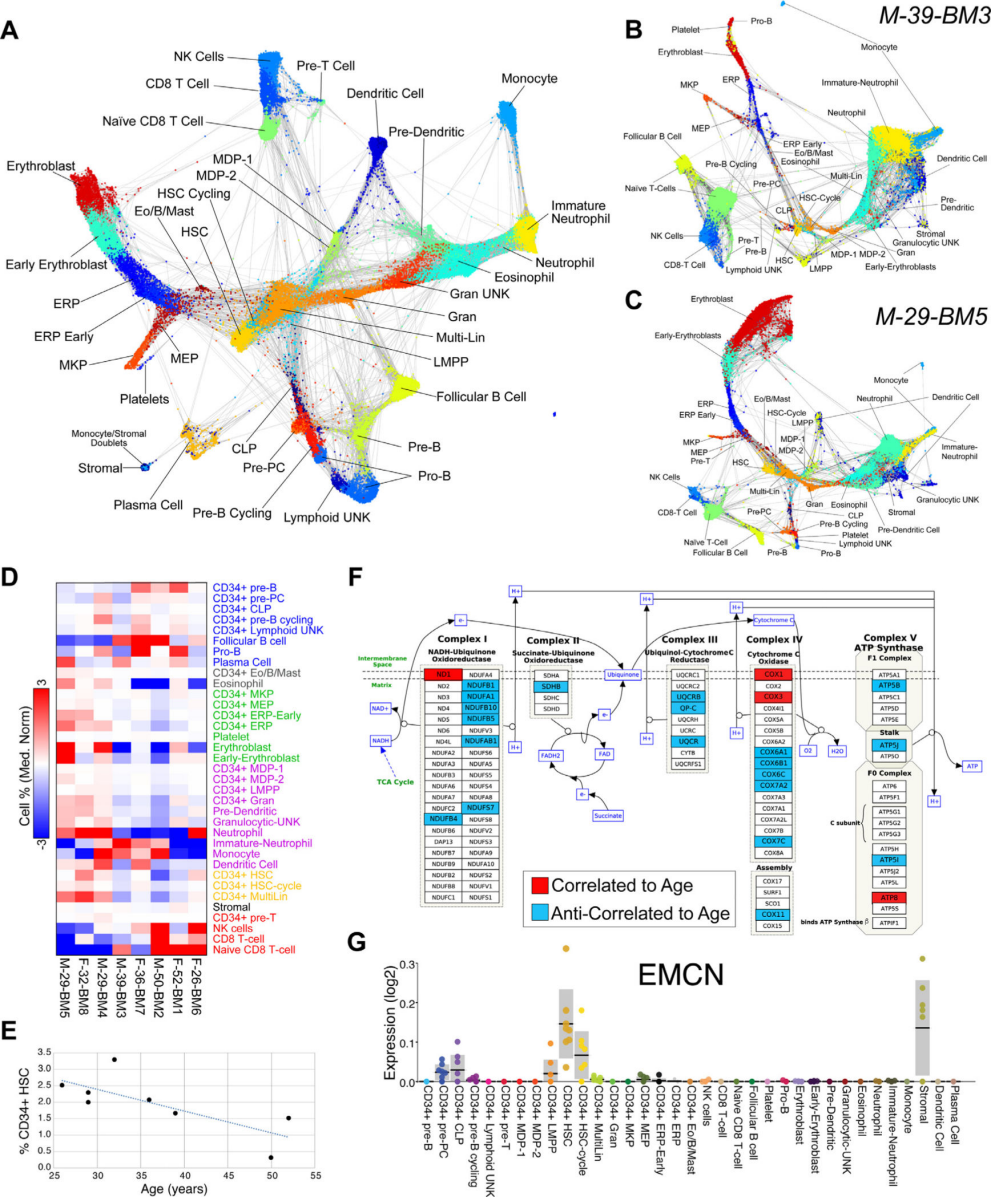
Cell populations and genes covary among donors. **(A)** SPRING analysis of all combined populations (top 1,500 cellHarmony-ranked cells per population). Dual lineage-associated cell populations (e.g., stromal) are predicted to be grouped with other lineages. **(B,C)** SPRING analysis of individual donors with varying cell population frequencies (immature neutrophils and erythroblasts). Population colors match panel **(A). (D)** Relative differences in population frequencies between donors organized according to annotated lineages (**y**-axis). **(E)** HSC cell frequency anticorrelation with age. **(F)** Visualization of correlated (red) and anticorrelated (blue) genes in HSC with donor age (rho > 0.6 or rho < −0.6) upon the Electron Transport Chain WikiPathway (WP111). **(G)** Variation in the expression of the prior proposed human HSC marker gene EMCN in our HCA web viewer.

To obtain a global view of cells from all donors, we combined the resolved progenitor and committed cell population predictions for SPRING analysis of the associated marker genes (Figure 3A, Supplementary Table E5, online only, available at www.exphem.org). These results confirmed and extended our progenitor trajectory predictions. As noted from our preliminary analysis (Figure 1A), different individuals from this HCA bone marrow dataset display differences in the frequencies of different hematopoietic cell populations. Restricted analysis of individual donors using SPRING highlights large differences in the presumed production of distinct lineages in different donors (Figures 3B and 3C). For example, donor 3 accounts for ~40% of all “immature neutrophils” in the combined dataset, but contains relatively few erythroblasts, with a somewhat deformed SPRING graph, inconsistent with the combined donor version and a representative donor (donor 5).

Comparison of cell population frequencies among donors further supports the finding that differences in cell frequency are lineage dependent rather than technical (random cell sampling) (Figure 3D, Supplementary Table E6, online only, available at www.exphem.org). Specifically, we find that the frequency of HSC and Multi-Lin progenitors are correlated with each other, in addition to MEP, early ERP, LMPP, predendritic cells, MDP, Gran, and mature neutrophils (rho > 0.6). The frequencies of naive and mature T cells are also correlated with each other but anti-correlated with MDP2, LMPP, erythroblasts, eosinophil progenitors, predendritic cells, dendritic cells, and Multi-Lin. CD34^+^ pre- B cells display the largest variation in population frequency, ranging from 0.05% to 2.15% of all cells, and are correlated with the frequency of unknown CD34^+^ lymphoid progenitors and pro-B cells. Therefore, these patterns suggest differences in the production of distinct lineages in early progenitors that manifest in differentiated end states.

Although the initial HCA bone marrow dataset only contains eight donors, we attempted to assess differences in these donors associated with both sex and age relative to gene expression. Rather than providing concrete conclusions, due to the insufficient number of samples, these data can be used to identify preliminary associations for hypothesis generation. For gene expression analyses, we calculated average expression across all cellHarmony cell-population-classified cells (pseudobulk) to compare these values among donors. Comparison of pseudobulk expression between male and female donors among all 35 cell populations identifies minimal differences, which are attributed only to X-chromosomal genes (*XIST, RPS4X*) and Y-chromosomal genes (*RPS4Y1, EIF1AY, DDX3Y)*. Although over 200 genes are differentially expressed between males and females in platelets, these differences are attributed to the extremely small number of cells captured (*n* = 65) (not shown). In addition to gender, donors vary by age, ranging from 26 to 52 years. HSC fitness is a significant focus of regenerative medicine and aging, with decreased frequency of functional HSCs occurring over a human lifetime. During aging, the number of phenotypically identified HSC increases [21–25], whereas the number of functional HSC decreases [26] (Figure 3E). The cell frequency of HSC in this dataset decreased with age (Pearson rho= −0.75). Correlation of pseudobulk gene expression profiles in HSC among the eight donors finds 242 genes that are further anti-correlated with age and 139 that are correlated (Supplementary Table E7, online only, available at www.exphem.org). HSC-expressed genes anti-correlated with age are highly enriched in electron transport chain and proteasome degradation, whereas both correlated and anti-correlated transcripts are enriched among cytoplasmic ribosomal proteins and mRNA processing pathways (Figure 3F, Supplementary Table E7, online only, available at www.exphem.org). Two of the most highly correlated genes with age are *OLA1* (encoding the DNA Damage-Regulated Overexpressed on Cancer 45 Protein) and the myelodysplastic syndrome/acute myeloid leukemia-associated gene NSD3. These results are consistent with the possible role of decreased mitochondrial fidelity and increased risk of bone marrow failure and myeloid malignancy with age. We further observed variability in the expression of other genes, such as previously defined cell-population-specific markers (e.g., EMCN) from these data (Figure 3G).

### Interactive web browser

**Figure 4.**
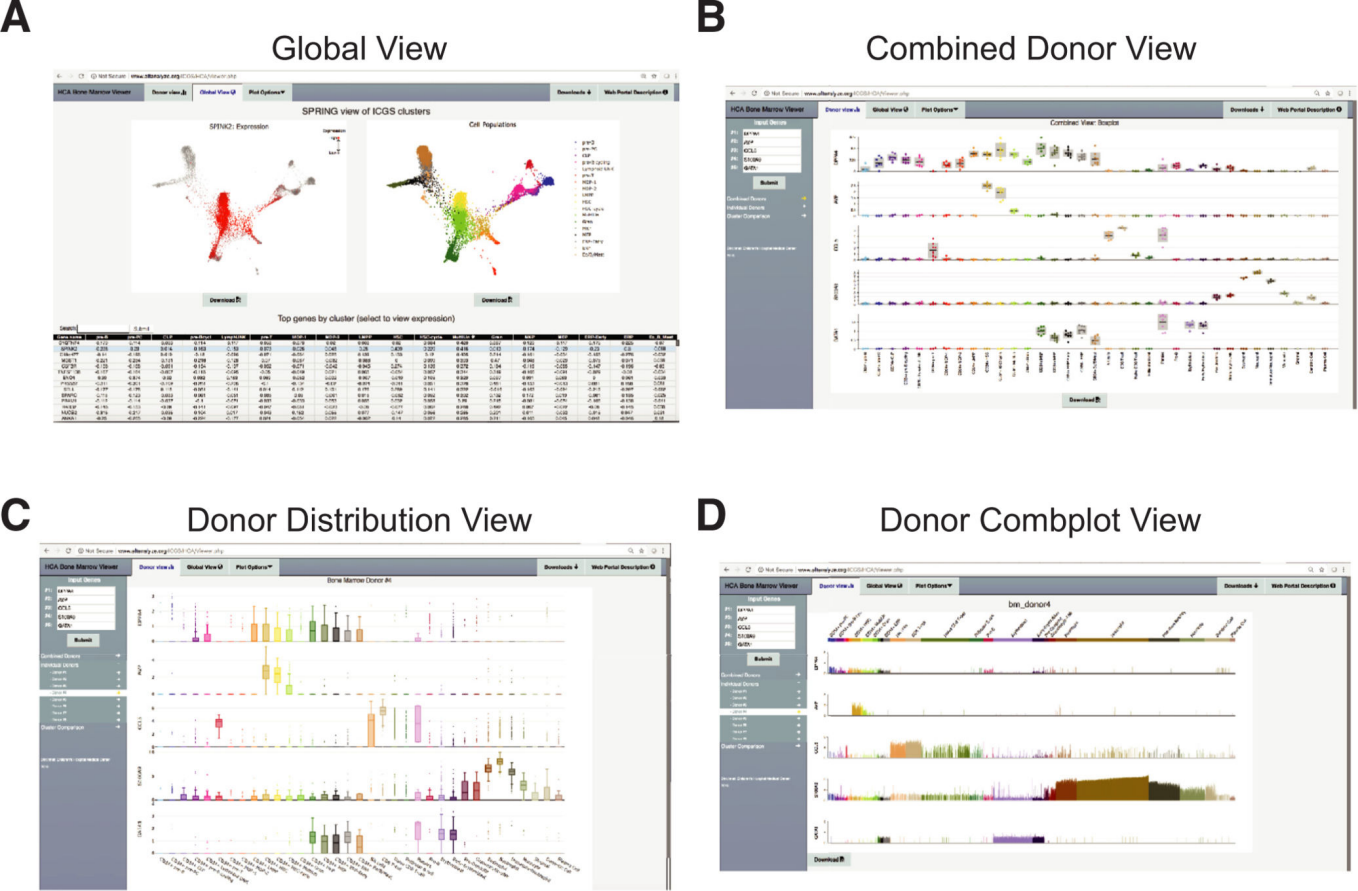
Interactive web portal for the HCA bone marrow data. Visualization options within the HCA bone marrow browser. **(A)** Global view for interactive visualization of the CD34^+^ cell SPRING analysis. Genes can be directly queried in the interface or identified from the list of cell- population-specific markers (sortable table). Each dot represents a single cell and specific cell populations can be hidden or exposed. **(B)** Combined donor view displaying the boxplot of the mean expression of each gene for each donor for each specific cell population. The donor information associated with each data point can be determined by mouse-over. **(C,D)** Individual donor expression variation viewed as data points for each individual cell as a **(C)** boxplot or **(D)** a bar chart to evaluate the frequency and amplitude of gene expression in all eight donors. New genes can be selected through the interface on the left. Source files can be downloaded through the interface (downloads tab).

To permit broad exploration and reanalysis of these identified cell populations, we developed an interactive data portal organized into three hierarchical views: (1) global cell population heterogeneity view (global view), (2) combined donor averaged gene expression profiles for all cell populations (combined donor view), and (3) individual donor-cell gene expression (individual donor view) (Figure 4). In the global view, users can query the expression of any gene across the spectrum of identified progenitor and differentiated cell populations, for all donors, distributed graphically as a SPRING or UMAP graph (Figure 4A). All genes in the database are included in a dynamically loading table. Genes with expression restricted to each cell population can be quickly obtained using the built-in table sort function of this view, which reports MarkerFinder specificity (Pearson correlation) scores (values close to 1 indicate highly restricted cell population expression) for all of the cell populations.

To explore common donor gene expression variation for any of the 35 defined cell populations, the user can switch to the combined donor view, which plots the variation in expression among the eight donors for pseudobulk expression values (Figure 4B). Mousing over any data point indicates the sample meta-data. Users can switch between both box-plot and frequency bar chart (combplot) views of these data. To independently explore and visualize the frequency, distribution, and amplitude of gene expression in individual donors, the user can switch to the individual donor view (Figures 4C and 4D). As an example, users can compare the distribution of expression of individual cells within the HSC in young versus middle-aged donors to assess consistency in expression.

## Discussion

Despite decades of genomics analyses investigating genetic and genomic heterogeneity underlying human tissue biology, a cellular census of human heterogeneity that can be related to genetics has been lacking. The creation of human cellular reference maps that span the continuum of progenitor specification will provide essential insights into the cell-population-specific expression of genes and how that process varies across donors with distinct genes and over donor lifespan. We present a comprehensive global view of hematopoietic diversity derived by analysis of over 100,000 cells from the HCA project, providing new insights into variation of cell populations across individuals, ultra-rare and mixed-lineage primed progenitor states, and age-related changes in cellular frequency and gene expression. This work complements other ongoing efforts to develop interactive web platforms for the exploration of single-cell hematopoietic diversity [20,27]. In addition to these significant efforts, the current web portal provides the capability to explore transcriptomic variation among human donors that vary by sex, age, and hematopoietic population frequency. Such data represent a significant knowledge base as well as numerous testable hypotheses that cannot be sufficiently mined from this current analysis alone. By itself, this resource enables the identification of new markers distinct to or shared between individual cell populations. For example, new markers of CD34^+^ progenitor populations can be readily identified and may serve as improved selection markers for progenitor cell isolation (e.g., DPPA4) (Figure 4B). Our analysis identifies significant differences in the production of distinct lineages among different donors, in which the frequency of early progenitor cells are correlated with later committed precursors such as erythroid precursors and erythroblasts. Although a focus of the current work has been on heterogeneity within bone marrow hematopoietic progenitors (CD34^+^), similar downstream analyses of these and other cell populations are likely to resolve additional cell states not described here. Whereas we are reasonably confident that our combined donor analysis accurately classifies cells into comparable cell populations, confirmation will ultimately require definitive independent experimental analyses. To facilitate broad reuse and evaluation of our predictions, we have focused this work on the development of a highly accessible bioinformatics platform to navigate this large dataset of over 100,000 cells spanning eight healthy individuals.

Although this resource only includes eight healthy adult donors at present, which limits the analytical interpretation of sex, age, and cellular variations, the associated data represent a meaningful starting place for identifying consistent effects in larger orthogonal and newly developed resources. Nonetheless, we observed outliers in these data that provide new insights into intradonor cellular variation, such as the excessive production of our annotated immature neutrophils coincident with a loss of erythroid precursors (Figure 3B). Although such results are not entirely definitive because the data represent only a single snapshot in time, they suggest that the immunological repertoire of at least one donor is highly skewed, similar to that expected in anemia of inflammation, iron deficiency, and/or emergency granulopoiesis. Other examples include genes that change in expression within specific cell populations with age that predispose to hematological malignancies such as myelodys-plastic syndrome or acute myeloid leukemia. Our analysis highlights the power of an open-access HCA, in which new analytical interfaces can be developed by the larger research community. We believe that this resource represents a unique source for hypothesis evaluation that we aim to expand over time to gain improved insights into progenitor specification trajectories, lineage potential, and associated variation in the human population.

## Supplementary Material

1

2

3

4

5

6

7

8

9
