# Supplementary figures and images for: The Human Cell Atlas bone marrow single-cell interactive web portal
Source: Exp Hematol. Author manuscript; Available in PMC 2019 Dec 1. (PMC6296228; doi:10.1016/j.exphem.2018.09.004)

**Figure S1**

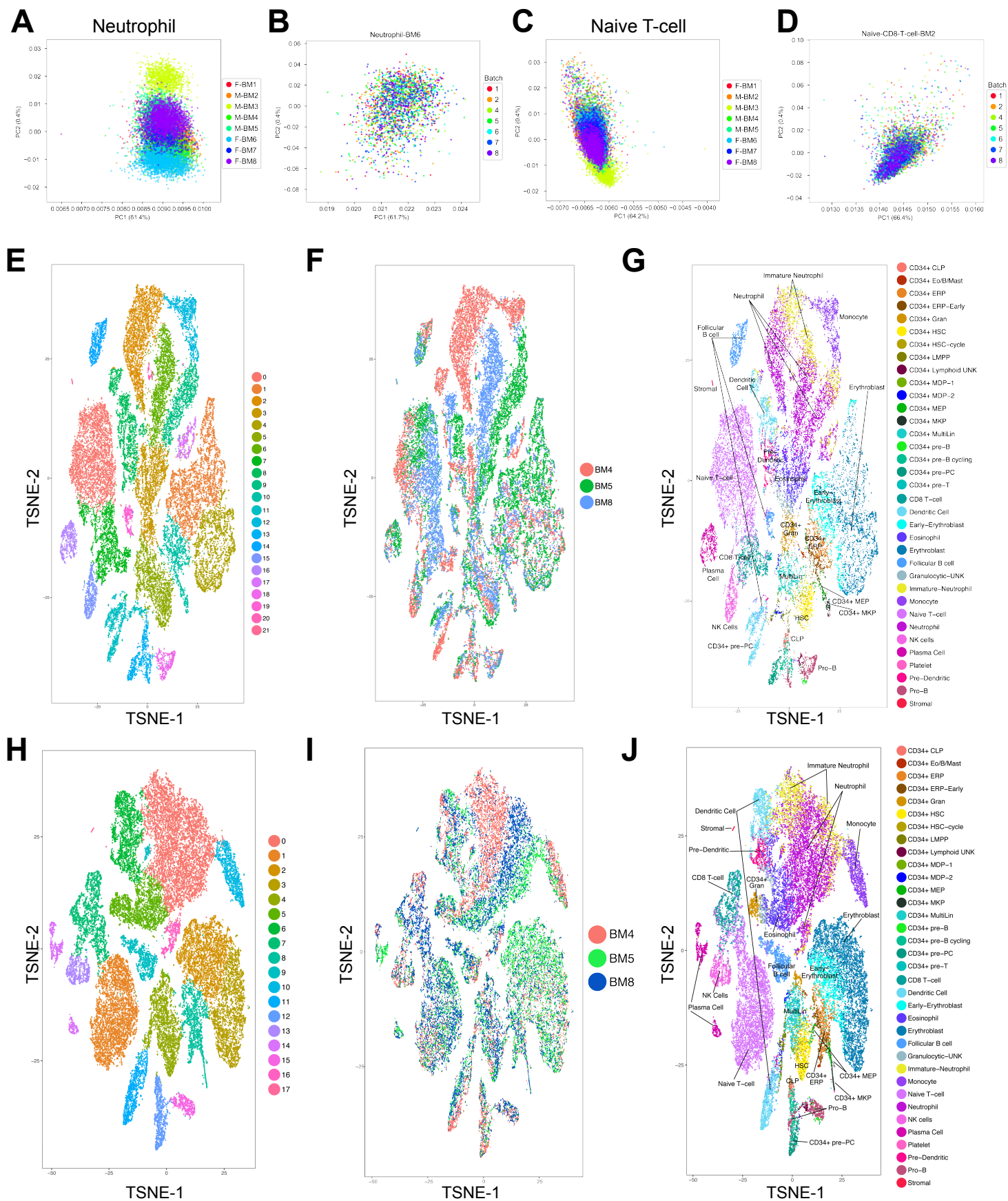

Supplement: 1 [file NIHMS1510309-supplement-1.pdf]
