# Supplementary material for: The Human Cell Atlas bone marrow single-cell interactive web portal
Source: Exp Hematol. Author manuscript; Available in PMC 2019 Dec 1. (PMC6296228; doi:10.1016/j.exphem.2018.09.004)
Supplement: 2 [file NIHMS1510309-supplement-2.docx]

### Human Cell Atlas Immune Analysis Workflows

# Below, find the commands used to run the workflow described in Hay et al.

# Where indicated, steps were performed using an LSF manager (BSUB) due to dataset size, but are not required.

# Download AltAnalyze from Github (https://github.com/nsalomonis/altanalyze)

# Install necessary dependencies (Python 2.7, R, Scipy, Numpy, Matplotlib, Tkinter)

# Install the human Ensembl 72 database

python AltAnalyze.py --species Hs --update Official --version EnsMart72 --additional all

# Download the gene counts quantified gene expression

# https://preview.data.humancellatlas.org

# Normalize expression values from counts for all cellular barcodes

#BSUB -n 4

#BSUB -M 256000

module load python/2.7.5

python import_scripts/DropSeqProcessing.py --i $PATH

# Filter normalized expression file for barcodes with at least 200 genes expressed

#BSUB -n 4

#BSUB -M 64000

module load python/2.7.5

python import_scripts/sampleIndexSelection.py --i $PATH --geneCountFilter TRUE --expressionCutoff 1 --minGeneCutoff 199

# Perform ICGS on each individual donor samples (8 independnet batches combined) - script is run separately for ech donor text file

#BSUB -n 4

#BSUB -M 128000

module load R/3.2.2

module load python/2.7.5

python AltAnalyze.py --runICGS yes --platform "RNASeq" --species Hs --column_method hopach --column_metric euclidean --rho 0.3 --ExpressionCutoff 1 --FoldDiff 4 --SamplesDiffering 4 --restrictBy protein_coding --excludeCellCycle conservative --expdir $PATH --output $DIR

# Create a combined cellHarmony reference file with unique cell-state references from the 8 MarkerFinder (MF) references

#BSUB -n 4

#BSUB -M 128000

module load R/3.2.2

module load python/2.7.5

python AltAnalyze.py --species Hs --platform RNASeq --cellHarmonyMerge yes --o $OUTPATH --i $BM1MFPATH --i $BM2MFPATH --i $BM3MFPATH --i $BM4MFPATH --i $BM5MFPATH --i $BM6MFPATH --i $BM7MFPATH --i $BM8MFPATH

# Perform cellHarmony on the combined donor dataset (100k cells) - REFPATH is the result from cellHarmonyMerge

#BSUB -n 4

#BSUB -M 128000

module load python/2.7.5

python AltAnalyze.py --species Hs --platform RNASeq --cellHarmony yes --correlationCutoff 0.7 --reference $REFPATH --input $BMPATH

# Filter and sort the combined Bone Marrow expression file with the produced groups file (groups.cellHarmony.txt)

#BSUB -n 4

#BSUB -M 128000

module load python/2.7.5

python import_scripts/sampleIndexSelection.py --i $PATH --f $CHPATH

# Perform MarkerFinder analysis on the cellHarmony filtered results (move to ExpressionInput dir, renamed to exp.cellHarmony.txt, create comps.cellHarmony.txt with at least one group comparison)

#BSUB -n 4

#BSUB -M 128000

module load python/2.7.5

python AltAnalyze.py --species Hs --platform RNASeq --update markers --expdir $BMPATH

# Filter all cells for selected CD34+ clusters (use the sampleIndexSelection.py script as shown above)

# Perform ICGS on the CD34+ selected cell populations as shown above

# Identify mixed-lineage marker genes (requires groups ordered expression file)

python stats_scripts/multiLineagePredict.py --expdir $ExpFile --m $MarkerFinderDir

# Run SPRING analysis from python command-line

import pickle, numpy as np

from preprocessing_python_gl import *

E, gene_list = importTextFile('expression_file.txt')

E = row_normalize(E)

# Filter genes with mean expression < 0.1 and fano factor < 3

print 'Filtering genes',gene_filter = filter_genes(E,0.1,3)

# Z-score the gene-filtered expression matrix and do PCA with 20 pcs

Epca = get_PCA(Zscore(E[:,gene_filter]),20)

# get euclidean distances in the PC space

D = get_distance_matrix(Epca)

cell_groupings = importCellGroupings('groups_file.txt')

# save a SPRING plots with k=5 edges per node in the directory

save_spring_dir(E,D,5,gene_list,'datasets/output_file', cell_groupings=cell_groupings)

# Run UMAP from python command-line

import scanpy.api as sc

import pandas as pd

import matplotlib.pyplot as plt

import umap

# Read a data file to an AnnData object (MarkerFinder population-specific genes)

filename='expression_file.txt'

expMatrix = pd.read_csv(filename,sep='\t',header=0,index_col=0).T

embedding = umap.UMAP().fit_transform(expMatrix)

plt.scatter(embedding[:, 0], embedding[:, 1], s=0.1, cmap='Spectral')

umapCoordinates = pd.DataFrame(data=embedding,index=expMatrix.index.values.tolist(),columns=['UMAP_1','UMAP_2'])

umapCoordinates.to_csv('umap_output.txt',sep='\t')
